# Longevity in Mice Is Promoted by Probiotic-Induced Suppression of Colonic Senescence Dependent on Upregulation of Gut Bacterial Polyamine Production

**DOI:** 10.1371/journal.pone.0023652

**Published:** 2011-08-16

**Authors:** Mitsuharu Matsumoto, Shin Kurihara, Ryoko Kibe, Hisashi Ashida, Yoshimi Benno

**Affiliations:** 1 Dairy Science and Technology Institute, Kyodo Milk Industry Co. Ltd., Tokyo, Japan; 2 Benno Laboratory, Innovation Center, RIKEN, Wako, Japan; 3 Division of Applied Biology, Graduate School of Science and Technology, Kyoto Institute of Technology, Kyoto, Japan; 4 Division of Integrated Life Science, Graduate School of Biostudies, Kyoto University, Kyoto, Japan; Fundació Institut Germans Trias i Pujol; Universitat Autònoma de Barcelona CibeRES, Spain

## Abstract

**Background:**

Chronic low-grade inflammation is recognized as an important factor contributing to senescence and age-related diseases. In mammals, levels of polyamines (PAs) decrease during the ageing process; PAs are known to decrease systemic inflammation by inhibiting inflammatory cytokine synthesis in macrophages. Reductions in intestinal luminal PAs levels have been associated with intestinal barrier dysfunction. The probiotic strain *Bifidobacterium animalis* subsp. *lactis* LKM512 is known to increase intestinal luminal PA concentrations.

**Methodology/Principal Findings:**

We supplemented the diet of 10-month-old Crj:CD-1 female mice with LKM512 for 11 months, while the controls received no supplementation. Survival rates were compared using Kaplan–Meier survival curves. LKM512-treated mice survived significantly longer than controls (*P*<0.001); moreover, skin ulcers and tumors were more common in the control mice. We then analyzed inflammatory and intestinal conditions by measuring several markers using HPLC, ELISA, reverse transcription-quantitative PCR, and histological slices. LKM512 mice showed altered 16S rRNA gene expression of several predominant intestinal bacterial groups. The fecal concentrations of PAs, but not of short-chain fatty acids, were significantly higher in LKM512-treated mice (*P*<0.05). Colonic mucosal function was also better in LKM512 mice, with increased mucus secretion and better maintenance of tight junctions. Changes in gene expression levels were evaluated using the NimbleGen mouse DNA microarray. LKM512 administration also downregulated the expression of ageing-associated and inflammation-associated genes and gene expression levels in 21-month-old LKM512-treated mice resembled those in 10-month-old untreated (younger) mice.

**Conclusion/Significance:**

Our study demonstrated increased longevity in mice following probiotic treatment with LKM512, possibly due to the suppression of chronic low-grade inflammation in the colon induced by higher PA levels. This indicates that ingestion of specific probiotics may be an easy approach for improving intestinal health and increasing lifespan. Further studies are required to clarify its effectiveness in humans.

## Introduction

More than 100 years have passed since Metchnikoff introduced oral bacteriotherapy to prevent intestinal putrefaction and ageing [1]. However, no study has reported an increase in longevity following treatment with yogurt or probiotics.

Many mechanisms have been shown to contribute to the process of senescence, such as telomere shortening in replicative cells, cumulative damage to DNA leading to genomic instability, oxidative damage to critical molecules by reactive oxygen species (ROS), and so on [2]. These mechanisms also comprise chronic low-grade inflammation, a major risk factor for ageing and age-related diseases, such as Alzheimer's disease and type II diabetes [3], [4]. Furthermore, the prevention of chronic low-grade inflammation appears to be one of the most effective approaches for increasing longevity [5].

At least 1,000 bacterial species have been found to inhabit the human intestine, and 10^14^ individual bacterial cells of at least 160 different species inhabit each individual's intestine [6], which is 10 times greater than the total number of somatic and germ cells in the human body [7]. Intestinal microbiota plays a fundamentally important role in health and disease [7]. In the healthy intestinal tract, the microbiota and the gut-associated immune system are assumed to share a fine and dynamic homeostatic equilibrium [8]. The chronic low-grade inflammation process may undermine this balance. Although there have been no studies demonstrating the relationship between intestinal luminal environment, chronic low-grade inflammation , and lifespan, we believe that lifespan can be extended by the inhibition of chronic low-grade inflammation via the control of intestinal microbiota for the following 4 reasons. First, the lifespan of germ-free mice is longer than that of conventional mice [9]; second, intestinal microbiota undergoes alterations by ageing [10], [11]; third, intestinal microbiota stimulates host mucosal and systemic immunity [12]; and lastly, ageing-associated deterioration in intestinal barrier functions may permit increased systemic absorption of intestinal luminal antigens [13].

Polyamines (PAs), such as putrescine (PUT), spermidine (SPD), and spermine (SPM), are organic cations required for cell growth, cell differentiation, and for the synthesis of DNA, RNA, and proteins [14]. PAs are known to possess anti-inflammatory activity via the inhibition of inflammatory cytokine synthesis in macrophages [15] and the regulation of NFκB activation [16], and are closely associated with the maintenance of the intestinal mucosal barrier function [17]. Furthermore, PAs function as ROS scavengers, acid tolerance factors, chemical chaperones, and positive regulators for the expression of stress response genes [18]; PAs also have antimutagenic activity [19]. As exogenous PAs derived from meals are absorbed before they reach the lower parts of the intestine [20], it has been suggested that the greatest amounts of the PAs in the lower parts of the intestine are synthesized by intestinal microbiota [21]. In mammals, body PA levels decrease during the ageing process [22]; intestinal PA concentrations in the elderly are lower than those in healthy adults [23], suggesting that these compounds may be linked to senescence and longevity.

The probiotic strain *Bifidobacterium animalis* subsp. *lactis* LKM512 (hereafter referred to as LKM512) exhibits potent acid tolerance and the ability to adhere to intestinal mucin, alters intestinal microbiotic populations [24], [25], and increases intestinal PA concentrations in humans [24], [26]. In fact, consumption of yogurt containing LKM512 has been shown to result in increased intestinal PA concentrations and decreased levels of acute inflammation markers in hospitalized elderly patients [26].

On the basis of these data, we hypothesized that the use of probiotics such as LKM512 would increase mammalian longevity by suppressing chronic low-grade inflammation [27]. To test these hypotheses, we examined middle-aged (10-month-old) mice that were provided a standard diet supplemented by oral doses of LKM512 or control. After 6 months of treatment (when the mice were 16 month old), LKM512 mice showed increased longevity as compared to the control mice.

## Results

### Effects of LKM512 treatments on survival

**Figure 1:**
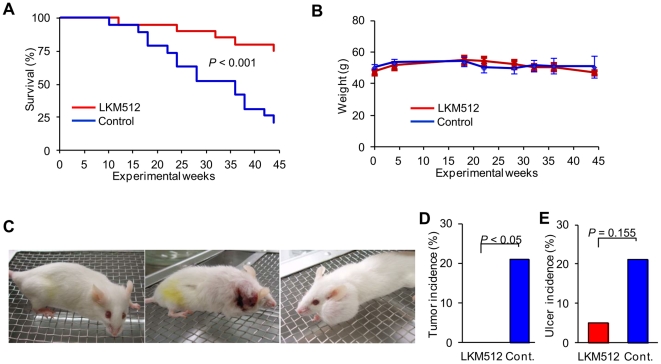
Impact of LKM512 on lifespan, weight, and appearance. (A) Kaplan–Meier survival curves. (B) Weight differences between treatment groups during the study period. (C) Typical appearance of 20-month-old mice. LKM512 mice appeared healthy (left), but many control mice had skin ulcers (middle) and tumors (right). Incidence of visible tumors (D) and skin ulcer (E) in the different treatment groups.

Mice treated with LKM512 lived significantly longer than controls (*P*<0.001) (Fig. 1A). The survival curves of the 2 groups began to diverge at 20 weeks (when the mice were 15 months old), and remained apart until the end of the study. However, there was no significant weight difference between the groups, or any noticeable difference in weight fluctuation during the study period, although mice in both treatment groups were given a standard pellet chow diet *ad libitum* (Fig. 1B). It is important to note that the longevity observed in the LKM512-treated mice was not related to calorie restriction (CR), which has been shown to increase longevity in a variety of species [28].

In the mice in the control group, the incidence of visible tumors (*P*<0.05) and skin ulcers (*P* = 0.155) was higher than that in the LKM512 treatment group (Fig. 1C–E). Additionally, skin and hair quality was better in LKM512 mice than in control mice, indicating that this treatment allows mice to not only live longer, but also be healthier as they age. These results were not influenced by the stress of establishing and maintaining social rank, because we observed no fighting amongst the female mice used in this study.

### Response of intestinal environment to LKM512

**Figure 2:**
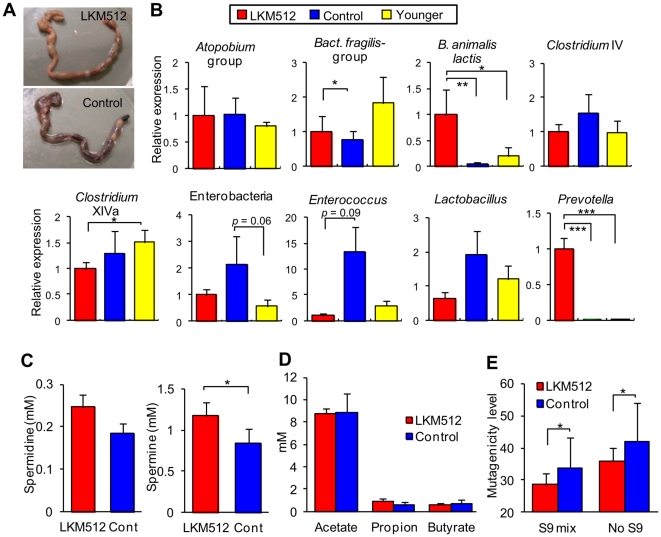
Influence of LKM512 administration on intestinal environment. (A) Appearance of large intestine obtained from LKM512 (upper) and control (bottom) mice at week 45. (B) 16S rRNA gene expression in the predominant intestinal bacterial group at week 45. 16S rRNA gene expression in the bacterial group was normalized to total bacterial 16S rRNA expression. Expression in control and younger mice is shown relative to the expression in the LKM512 mice. (C) Fecal SPD (right) and SPM (left) concentrations in each group. (D) The concentration of SCFA in each group. (E) Fecal mutagenicity stimulated with S-9 mix and without S-9 mix in each group. In (B) – (E), data are represented as mean ± SEM. *P<0.05, **P<0.01, ***P<0.001.

LKM512 administration inhibited constipation; however, the large bowel contained many feces with low water content (50–60%) in half of the control mice that survived until week 45 (21 months old). In contrast, the large bowel of LKM512-treated mice contained feces with high water content (60–70%) (Fig. 2A). This suggests that the control mice might have displayed symptoms of constipation.

We used reverse transcription-quantitative PCR (RT-qPCR)to quantify commensal gut bacteria by measuring their expression of the 16S rRNA gene. In general, we found that the LKM512 treatments altered 16S rRNA gene expression of several predominant bacterial groups by week 45 (Fig. 2B). Specifically, the 16S rRNA gene expression of both *B. animalis* subsp. *lactis* (LKM512) and *Prevotella* spp. were detected in all mice from the LKM512 group and were higher than that in control mice (*P<*0.01 and *P*<0.001, respectively). To the best of our knowledge, this is the first demonstration that *Prevotella* counts may be increased by the administration of probiotics. Furthermore, these values were higher at the end of the LKM512-treatment period when the mice were 21 months old, than before the treatment began when mice were 10 months old (younger mice) (*P*<0.001). Following LKM512 treatment, 16S rRNA gene expressions of *Clostridium* cluster XIVa (vs. younger mice, *P*<0.05) were also altered. We also found evidence that LKM512 administration suppressed age-related changes in intestinal microbiota: 16S rRNA gene expression of the *Bacteroides fragilis* group (*P*<0.05) and Enterobacteriaceae species (*P* = 0.06) differed between the control group and younger mice, but not between the LKM512-treated and younger mice. Other bacterial genera showed no alterations.

The fecal concentrations of PAs, particularly SPM, which is the most bioactive PA, were significantly higher in the LKM512 mice than in the control mice (*P*<0.05, Fig. 2C). The correlation between fecal SPM concentration and 16S rRNA gene expression of the predominant bacterial group is shown in Fig. S1. This may have been due to the activity of *B. animalis* subsp. *lactis* (LKM512) and/or *Prevotella* spp., caused by the administration of LKM512, since the SPM concentration correlated better with the 16S rRNA gene expression of these bacteria as compared to other bacterial groups (Fig. S1). However, in this study, we could not specifically identify the bacterial group that produces PAs. Moreover, the complete genome sequence for *B. animalis* subsp. *lactis* indicates that this species does not possess the pathways for SPM synthesis; therefore, it was clear that LKM512 itself did not produce SPM. LKM512 administration did not significantly change short chain fatty acids (SCFA) concentrations (Fig. 2D). Fecal mutagenicity was significantly lower in the LKM512 mice than in control mice (*P*<0.05, Fig. 2E). PAs are known to have strong bioantimutagenic activities [19], we considered that this might be due to the increase in SPM concentration caused by LKM512 administration. However, other factors may have been involved in decreasing mutagenicity, e.g., binding of the mutagen to LKM512 cell walls as well as those of other bifidobacterial strains [29] or a decrease in mutagens caused by enzymes derived from the altered intestinal microbiota, such as β-glucuronidase.

### Effects of LKM512 on colonic barrier function

**Figure 3:**
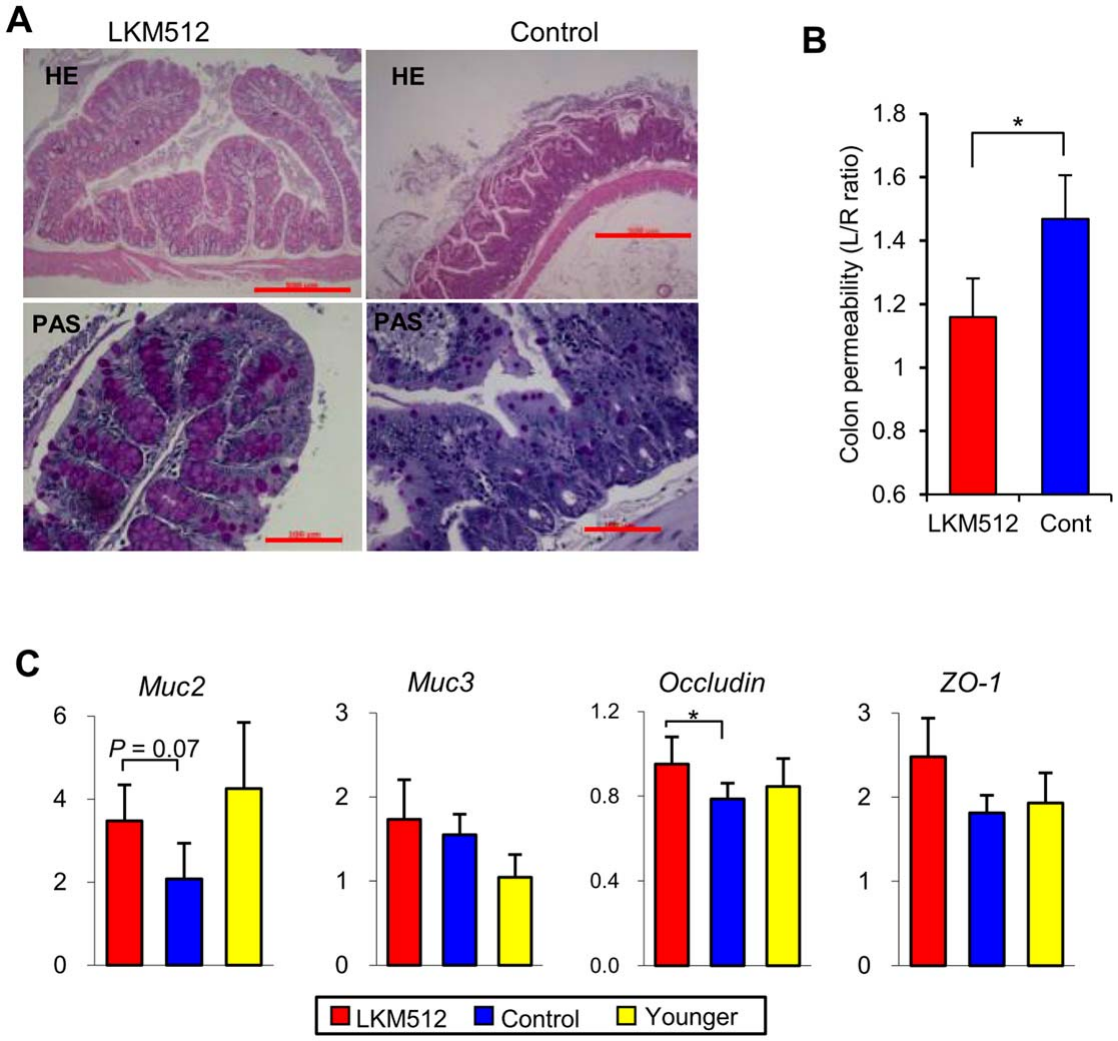
Influences of LKM512 administration on colonic barrier function. (A) Histology of proximal colon samples from mice treated with LKM512 (left) or PBS (control; right). The samples shown in the upper panels are stained with HE, while those in the bottom panels are stained with PAS. [Scale bars, 500 µm (HE), 100 µm (PAS).] (B) Colon permeability at week 25, as indicated by the urinary lactulose/rhamnose ratio. (C) Colonic gene expression of Muc2, Muc3, Occludin, and ZO-1 in all treatment groups and in younger mice. Data are represented as mean ± SEM. *P<0.05.

Mice that received LKM512 treatments had functional colonic mucosal layers; for instance, goblet cells were observed along the length of the crypt. On the other hand, there were clear signs of degradation in mucosal function in control mice, in whom the crypts were degraded and few goblet cells were observed in ∼50% of the control mice that survived until week 45 (21 months) (Fig. 3A). At week 25, the urinary lactulose/rhamnose (L/R) ratio of LKM512 mice was significantly lower than that in control mice (*P*<0.05) (Fig. 3B). This indicated improved maintenance of the intestinal barrier function by LKM512 treatment [30].

LKM512 treatment changed the gene expression of some intestinal barrier associated proteins (Fig. 3C). Administration of LKM512 tended to increase the expression of *MUC2*, a mucin that is secreted by goblet cells to form the colonic mucus layer (*P* = 0.07). Levels of *MUC2* gene expression in the LKM512 group were equivalent to those observed in younger mice. However, colonic gene expression of *MUC3*, a membrane-bound mucin, was not altered by LKM512 administration. Colonic gene expression of occludin, one of the tight junction-associated proteins that help maintain intestinal barrier function, was increased by LKM512 administration (*P*<0.05). However, zonula occludens (ZO)-1 was not altered by LKM512 administration.

### Colonic microarray analysis

**Figure 4:**
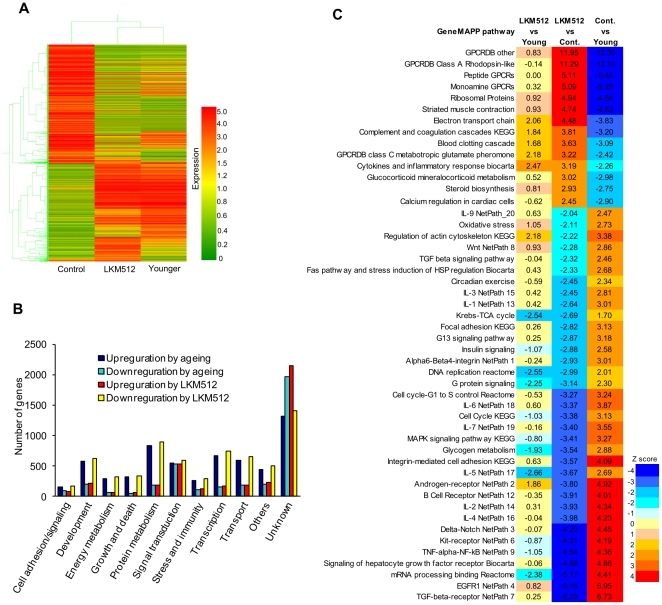
Microarray analysis of middle colon samples from LKM512-treated mice. (A) Hierarchical clustering showing patterns of expression relationships among LKM512-treated, control, and younger mice. Red and green indicate up- and downregulation of gene expression, respectively. (B) The number of genes up- and downregulated during the ageing process in each of the putative functional categories on microarray analysis. (C) Comparison of pathways that were significantly upregulated (red) and downregulated (blue) by LKM512 administration and ageing.

We examined gene expression patterns in the middle colons of mice in each of the 3 groups, i.e., LKM512 mice, control, and younger (pretreatment) mice (Fig. 4A). Using 4-fold change as a cutoff, we detected 11,164 differentially expressed genes. Although expression patterns in LKM512 mice were similar to those in younger mice, these patterns contrasted with that of control mice. This suggests that senescence-associated colonic gene expression was suppressed by LKM512 administration. By combining the functional annotations for these genes, we were able to assign multiple biological functions to certain genes and classify them into 9 putative functional categories. This allowed us to determine that, in most of these categories, a greater number of genes were upregulated than downregulated during the ageing process (Fig. 4B).

When the mice aged or were administered LKM512 treatments, 55 of 93 gene pathways were altered significantly (Z-score >1.98), while the gene expression for 78 of these 93 gene pathways was similar between younger mice and mice receiving LKM512 (Fig. 4C, Fig. S2). Pathways that were downregulated by ageing were upregulated by LKM512 administration and vice versa. In other words, LKM512 administration suppressed ageing-associated change in gene pathways.

### Inflammatory and oxidative stress responses

**Figure 5:**
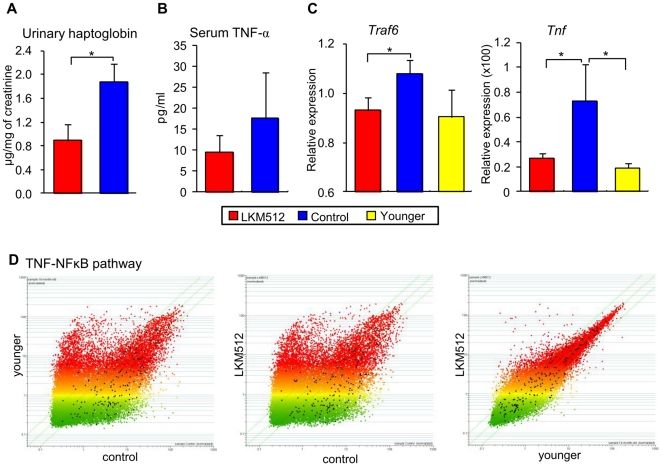
Influences of LKM512 and SPM administration on inflammation and oxidative stress. (A and B) Concentrations of urinary haptoglobin (A) and serum TNF-α (B). (C) Colonic gene expression of *Traf6* and *Tnf*. Data are expressed as the mean relative amount to mRNA of β-actin ± SEM. * *P*<0.05. (D) Microarray data scatter plots of the genes in the TNF-NFκB pathway. All genes (n = 25,631) are represented on scatter plots. The values for all the TNF-NFκB pathway genes represented on the array are highlighted in black. Younger (pretreatment) vs. control (left), LKM512 vs. control (middle), and LKM512 vs. younger mice (right).

We used urine haptoglobin levels to estimate intestinal inflammation [31]. At 25 weeks, haptoglobin levels were lower in LKM512 mice than in control mice (*P*<0.05) (Fig. 5A). Additionally, at 45 weeks, serum TNF-α concentrations tended to be lower in LKM512 mice than in control mice (Fig. 5B), as were gene expression levels of colonic *traf6* and *Tnf* (Fig. 5C) (*P*<0.05). These results indicate that LKM512 administration suppressed systemic and colonic inflammation caused by ageing. The anti-inflammatory effects of LKM512 administration were also revealed by a DNA microarray. Expression levels of genes in the TNF-NFκB, IL-1, IL-2, and IL-6 pathways were higher in the control group than in LKM512 and younger mice; additionally, gene expression levels in LKM512 mice were similar to those in younger mice (Fig. 5D and Fig. S3).

At 25 weeks, 8-OHdG concentrations, which indicate oxidative DNA damage [32], tended to be lower in LKM512 mice than in control mice (*P* = 0.09) (Fig. S4A). Furthermore, pathway analysis using a DNA microarray indicated that the oxidative stress pathway was more active in the control mice than in LKM512 mice (Fig. S4B).

## Discussion

In the present study, we have shown that the lifespan of mammals can be increased by probiotic treatment; furthermore, we have proposed that the mechanism by which this longevity was achieved is the suppression of chronic low-grade inflammation resulting from improvements in the intestinal luminal environment and the maintenance of colon tissue.

It is generally believed that intestinal bacterial components are recognized by Toll-like receptors under normal steady-state conditions, and this interaction plays a crucial role in host immunity [33]. Therefore, there is a possibility that this anti-chronic low-grade inflammation effect depends on the immunostimulation of intestinal microbiota altered by LKM512 treatment. However, our previous study demonstrated that the anti-inflammatory benefits of LKM512 are influenced by intestinal bacterial metabolites more directly than by immunostimulation due to bacterial cell components derived from certain strains within an altered colonic microbiota [34], [35]. There is also a possibility that orally administrated LKM512 cells directly stimulate lymphocytes in the Peyer's patches when they pass through the small intestine, similar to the effect of *Lactobacillus casei* strain Shirota [36], irrespective of the colonic luminal environment. Although this may be one of the mechanisms by which chronic low-grade inflammation was suppressed by LKM512 treatment, it cannot explain the inhibition of colonic inflammatory gene expression or the maintenance of colonic barrier function. We focused upon the fact that intestinal microbiota produce metabolites that have been shown to influence inflammation; for example, levels of PAs, which possess anti-inflammatory activity [15], [16] and the ability to maintain intestinal mucosal barrier functions [17], [37], were increased by LKM512 treatment.

**Figure 6:**
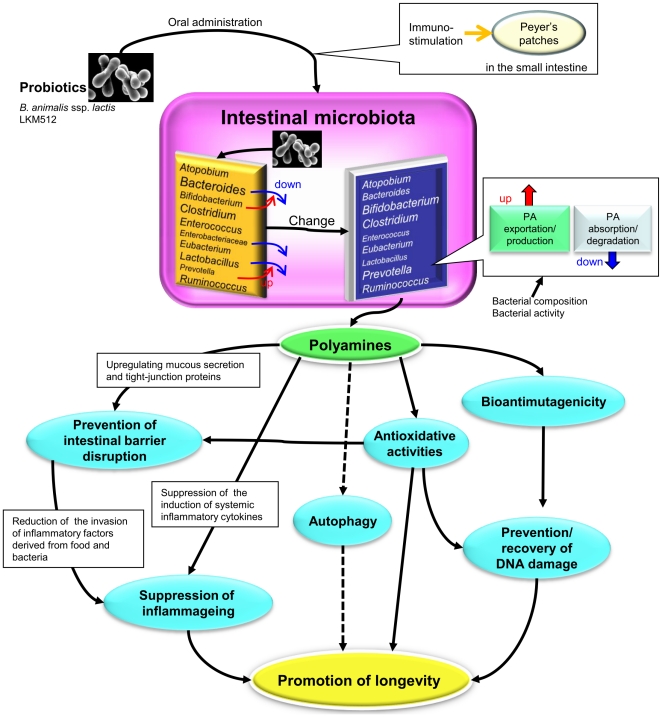
The hypothetical mechanism behind the increased lifespan of LKM512-treated mice (this is a modification of a part of a previous hypothesis [**27**]). After LKM512 is orally administered, it colonizes the colon and alters the intestinal microbiota, which then produces PAs. The alteration in intestinal microbiota by LKM512 facilitates the activity of *Prevotella* spp. but suppresses the *Bact. fragilis* group, Enterobacteriaceae species, and *Enterococcus* spp. The produced PAs induces maintenance and/or recovery of intestinal barrier function by upregulating mucous secretion; additionally, because of its antioxidative properties, it helps prevent colonic barrier disruption. Maintenance of the intestinal barrier reduces the intestinal inflammatory activity of factors derived from food and bacteria. Moreover, increased levels of PAs in the intestinal lumen lead to increased PA concentration in the blood. This circulating PA suppresses the induction and/or production of systemic inflammatory cytokines. At the same time, PAs possess bioantimutagenic and antioxidative activities that prevent DNA damage. Thus, the effects of PAs suppresschronic low-grade inflammation , thereby promoting longevity. Furthermore, although data is insufficient in this study, there is a possibility that autophagy induced by PAs also promotes longevity.

By focusing on PAs as principal bioactive substances, we proposed the hypothesis that increased intestinal levels of PAs would increase longevity by improving intestinal health and inhibiting systemic chronic low-grade inflammation (details in Fig. 6). Improved long-term survival has been demonstrated for SPD-treated cells and organisms [38], while PA-rich foods are known to decrease age-associated pathology and mortality in aged mice [39]. However, the oral administration of SPM did not improve longevity to the same extent as did the dose-intrinsic PAs supplied by colonic microbiota altered by LKM512 administration (*P* = 0.121) (Fig. S5A). This may be related to the quantity of PAs supplied: PAs delivered orally are transient, but PAs produced by colonic microbiota that have been enhanced by LKM512 treatment are continuously replaced. This allows for a much larger “dose” that is suitable for increasing longevity. This theory is supported by the differences in colonic SPM concentrations between LKM512 mice and SPM mice, wherein the SPM levels in SPM mice were as low as those in control mice (Fig. S5B), as well as by the differences in gene expression patterns (Fig. S5C). It is possible that other probiotic strains that have the ability to influence the intestinal environment, such as *B. bifidum* PRL2010—which metabolizes mucin derived from the host [40], and *Lactobacillus acidophilus* NCFM [41]—which increases intestinal PA levels following oral administration, may promote longevity. By using gnotobiotic mice singly associated with *Escherichia coli* O157, Fukuda et al. [42] demonstrated the protective abilities of *Bifidobacterium logum* JCM 1217^T^ against enteropathogenic infections through the production of acetate. However, acetate concentrations were not altered by LKM512 administration (Fig. 2D), suggesting that the promotion of longevity by LKM512 is not related to the protection against pathogenic bacterial infections by the acetate produced by LKM512 or other bacteria.

This study proposes a new finding concerning the relationship between the colon and antiaging. Cumulatively, treatment with LKM512 inhibited morphological disruption of the intestinal barrier during the ageing process [13], suggesting that maintenance of intestinal barrier function was one of the major factors in the promotion of longevity in this trial. Furthermore, LKM512 administration suppressed senescence of the colon by downregulating ageing-associated gene expression, rather than by upregulating rejuvenation-associated gene expression (Fig 4B). A report has associated modulation in gene expression through ageing with compromised intestinal function and propensity for colon cancer in the rat colon. We compared the changes in colonic gene expression in rats in that report [43] with our data (Table S1). In this study, we noted that almost all the genes upregulated by ageing in rats were similarly upregulated by ageing in mice; furthermore, these were downregulated by LKM512 treatment. It is interesting to note that the pathways (Fig. 4C; upper panel) that were upregulated by LKM512 administration and downregulated by ageing were G-protein-coupled receptor (GPCR)-related pathways. Currently, there is little information available regarding the relationship between GPCRs, intestinal microbiota, longevity, and/or PAs, although it is known that SCFA and GPCR interact to affect the inflammatory response [44]. Clearly, further studies are required to clarify these relationships. To the best of our knowledge, this is the first demonstration of anti-ageing in the colon by probiotics using global gene expression profiling. CR is known as one of the factors capable of increasing the lifespan in a variety of species [28]. CR-induced metabolic reprogramming may be a key event in the mechanism of lifespan extension [45]. Studies in yeast, worms, flies, and mice point to its influence on nutrient-responsive signaling molecules, including SIRT1, the mammalian target of rapamycin (mTOR), and proliferator-activated receptor-γ coactivator 1α (PGC-1α) [46]. On the other hand, lifespan extension has been demonstrated to be induced by the control of these signaling molecules even in the absence of CR, i.e., by the oral administration of rapamycin [47] and resveratrol [48]. Rapamycin reduces the expression of function of mTOR, which is a central regulator of many biological processes [49]. It is known that the genetic inhibition of TOR extends the lifespan in short-lived model organisms and mice [47]. In our present study, the mTOR pathway was not listed in the GenMAPP pathway analysis; however, since rapamycin suppresses the mTORC1 complex in the mTOR pathway, we analyzed genes downstream of mTORC1 (Table S2). Although 6 out of 10 genes were upregulated by ageing, 4 out of the 6 genes thus upregulated were downregulated by LKM512 treatment, indicating that one of the mechanisms of lifespan extension by LKM512 treatment may involve the suppression of the mTOR pathway, similar to the effect of rapamycin. Further research is warranted in order to confirm the phosphorylation levels of at least 2 substrates, i.e., P70-S6 kinase that phosphorylates S6 ribosomal protein and 4E binding protein 1, which is a key translational repressor protein.

Resveratrol, a pharmacological activator of SIRT1, can improve the lifespan and health of mice on a typical high-calorie diet [48]. Its effects lead to a decrease in the levels of insulin-like growth factor-1 (IGF-1), while AMP-activated protein kinase (AMPK) and peroxisome proliferator-activated receptor-γ coactivator 1α (PGC-1α) activity is increased [48]. However, in our present study, while LKM512 treatment caused only slight changes in IGF-1 gene expression, AMPK-related and PGC-1α gene expression were considerably decreased (Table S2), indicating that the mechanism underlying improved longevity by LKM512 treatment differed from resveratrol's mechanism. Recently, autophagy—the cellular process of cytoplasmic degradation and recycling—has been proposed to promote longevity [38]. Interestingly, all the above mentioned longevity-promoting regimens—including CR and inhibition of TOR with rapamycin, resveratrol, or PAs—have been associated with autophagy; in some cases, they have been reported to require autophagy for their effects [50]. In fact, colonic autophagy appeared to be promoted in LKM512 mice as compared to the controls, as revealed by LC3 conversion (Fig. S6).

At less than 36 weeks of treatment, the median survival of the control group was less than 19 months, which was considerably short in comparison to those reported in other longevity studies conducted in mice. For example, recent studies in C57BL/6 mice show control median lifespan of more than 850 d (28 months) [51], while longevity studies in the 4-way genetically heterogeneous mice show median lifespan of almost 900 d (30 months) [47]. Although there is a longevity study in which Crj:CD-1 mice show a control median survival of less than 18 months [39], further research is warranted to confirm this finding in at least 1 other strain of mice.

In the 1970s, it was reported that increases in PAs were related to neoplastic growth [52]. Since then, many researchers have regarded PAs as carcinogens. However, most of these historical studies were performed to test the effects of PAs on existing tumors or on the growth of tumors after the initiation of neoplastic growth [53], [54]. We have found no evidence that increased intake of PAs promotes oncogenic transformation in normal cells and animals. Furthermore, there are many reports that PAs are indispensable for normal functioning in a diverse group of organisms ranging from cells and bacteria to plants and mammals [55], [56]. We hope that the results presented here encourage more researchers to investigate the diverse and important bioactivities of PA.

## Materials and Methods

### Mice

**Figure 7:**
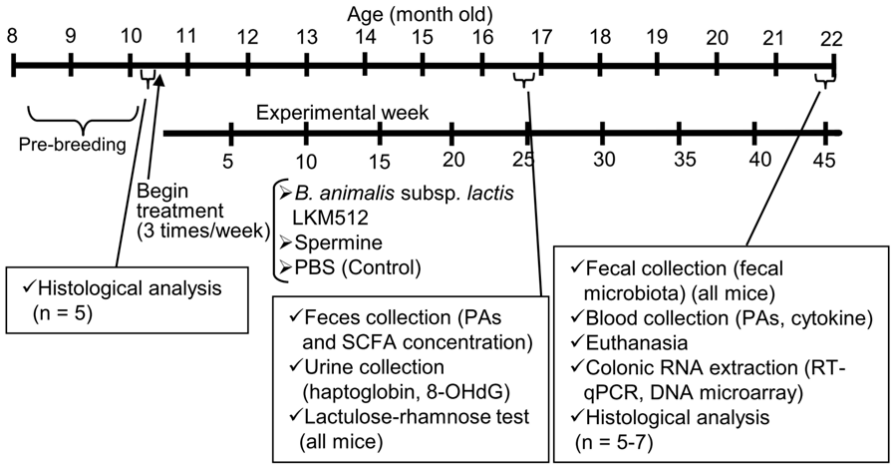
Experimental schedule. Female Crj:CD-1 (ICR) retired mice were obtained when they were 8 months old.

A closed colony of female 8-month-old Crj:CD-1 (ICR) retired mice (previously used solely as breeders) were obtained from Charles River Laboratories Japan Inc. (Kanagawa, Japan). Male retire mice could not be used because they are known to have violent tempers and to continuously fight with each other when kept in the same cage, which might have led to stress and its associated effects. Five mice were housed per cage in plastic cages (225×338×140 mm; Clea Japan, Inc., Tokyo, Japan) containing hardwood bedding. Mice were kept on a 12-h light/dark cycle at 25±1°C in conventional conditions. Mice were given a standard pellet chow diet *ad libitum* and gavaged with a 10 mM PBS solution containing *B. animalis* subsp. *lactis* LKM512 (n = 20), SPM (n = 20), or nothing (control treatment; n = 19). Treatment solutions were gavaged at 10 µL/g of body weight; LKM512 and SPM were administered at 10^9^ cfu⋅kg^−1^⋅dose^−1^ and 3 mg⋅kg^−1^⋅dose^−1^, respectively. This SPM content (mg⋅kg^−1^⋅dose^−1^ = approximately 0.15 mg⋅mice^−1^⋅dose^−1^) was calculated on the premise that half of the chow was comprised of soybeans, which are known to contain high levels of PAs. The standard pellet chow diet contained approximately 0.03 mg/g of SPM. LKM512 cells incubated on blood liver (BL) agar (Nissui Pharmaceutical, Tokyo, Japan) were harvested by swabbing and then suspended in PBS. The solution was administered 3 times a week, starting when the mice were aged 10 months. The experimental schedule is shown in Fig. 7. Weights were recorded for each mouse once every month throughout the study. Mice were examined at least daily for signs of ill health, and were euthanized if necessary. This experiment was performed in accordance with the protocols approved by the Kyodo Milk Animal Use Committee (Permit Number: 2005-03) and were in accordance with the Guide for the Care and Use of Laboratory Animals, published by the National Academies Press.

### Determination of fecal PA concentration

Fecal extracts were prepared as described in our previous report [24]. Fecal PA, PUT, SPD, and SPM levels were determined from fecal extracts with an Alliance 2695 HPLC system, using previously described methods [26]. Data analysis was performed using Empower 2 (Waters, Milford, MA).

### Determination of fecal water content

Fecal water content was calculated as the wet weight minus the dry weight of feces. The drying of feces was performed at 100°C for 3 h using a drying oven (ISIZU, Tokyo, Japan).

### Intestinal microbiota analysis by rRNA-targeted reverse transcription-quantitative PCR (RT-qPCR)

For RNA stabilization, frozen fecal samples were added to 10 volumes of RNA *later*-ICE (Applied Biosystems, Foster City, CA, USA) for at least 24 h. Total RNA was prepared by TaKaRa FastPure RNA Kit (Takara Bio Inc., Shiga, Japan) with on-column DNase treatment, following a modified version of the manufacturer's protocols. Each fecal mass was washed 3 times by suspension in 1.0 mL of PBS. Fecal pellets were resuspended in 500 µL of Lysis Buffer, and 200 mg of glass beads (diameter, 0.1 mm) were added. The mixture was treated at 70°C for 10 min in a water bath and vortexed vigorously for 60 s with a Micro Smash MS-100 (Tomy Digital Biology Co., Ltd., Tokyo, Japan) at 4,000 rpm. After centrifugation (14,000 *g*×5 min), 400 µL of supernatant was collected as a crude total RNA solution and purified following the manufacturer's protocols. Quantity and purity were confirmed by spectrophotometry (ratio  =  A_260_/A_280_). cDNA for each sample was synthesized using 250 ng of total RNA and a PrimeScript RT reagent kit (Takara), according to the manufacturer's instructions.

Real-time PCR for quantification of intestinal bacterial 16S rRNA gene expression was performed using the StepOne Real-time PCR system (Applied Biosystems) as described in our previous report [25] (Table S3). PCR reaction mixtures were composed of 100-fold diluted template cDNA. Fifty-fold, 500-fold, 5,000-fold, and 50,000-fold diluted fecal cDNA from the LKM512 group were used as the real-time PCR standard for all bacterial genera and groups. The 16S rRNA gene expression of each bacterial genera and group was normalized with the total bacterial value using a comparative delta Ct method; the expression in SPM, control, and younger mice was calculated relative to the expression in the LKM512-treated group.

### Determination of fecal SCFA concentration

Frozen fecal samples were diluted 10-fold with 2% perchloric acid, and soluble substances were extracted at 4°C for 18 h. After extraction, the precipitate was removed by centrifugation (10,000 *g*×10 min at 4°C) and the supernatant was filtered (0.45 µm) for use. SCFA levels were determined using a post-column HPLC setup, consisting of an Alliance 2695 HPLC System (Waters), a Shodex KC-811 with a guard column (Showa Denko, Tokyo, Japan), and a post-column color reaction with bromothymol blue reagent. Data analysis was performed using Empower 2.

### Lactulose/rhamnose intestinal permeability test

The L/R intestinal permeability test was performed as described in Koltun et al. [30], with some modifications. Mice were fasted overnight prior to being gavaged with a solution containing lactulose (1.5 g⋅kg^−1^) and rhamnose (0.3 g⋅kg^−1^) in distilled water. Urine was collected for at least 6 h in receptacles containing 200 µL of chlorohexidine (Sigma, St. Louis, MO, USA) as a preservative. Pretreatment was performed using a Sep-Pak Cartridge (Waters). Lactulose and rhamnose levels were determined using an Alliance 2695 HPLC system and a 2414 Refractive Index Detector (Waters). We used an ULTRON PS-80N (300×8.0 mm ID) column connected to an ULTRON PS-80NG (50×8.0 mm ID) guard column (Waters). The chromatogram analysis software used was Empower 2. Test results for the sugars were expressed as (peak area of lactulose)/(peak area of rhamnose).

### Determination of urinary haptoglobin concentrations

Haptoglobin was measured with mouse haptoglobin ELISA test kits (Life Diagnostics, Inc., West Chester, PA, USA) using 10-fold diluted urine. Haptoglobin was converted into the quantity of creatinine required for measurement by the QuantiChrom^TM^ Creatinine Assay Kit (BioAssay Systems, Hayward, CA, USA).

### Determination of urinary 8-OHdG concentrations

Urinary 8-OHdG was measured with 8-OHdG Check ELISA Kits (JaICA, Fukuroi, Japan) using 10-fold diluted urine. 8-OHdG was converted into the quantity of creatinine required for measurement with a QuantiChrom^TM^ Creatinine Assay Kit (BioAssay Systems).

### Determination of fecal mutagenicity

Fecal mutagenicity was determined with *umu*-tests conducted with commercial UMU-LAC kits (Jimro, Takasaki, Japan). As a standard in the fecal mutagenicity tests, we used 2-aminoanthracene (0.37–30 µg/mL) with S-9 mix. S-9 mix was used to test mutagenicity in the presence of metabolic activation. The mutagenicity level of each fecal extract was expressed as 2-aminoanthracene concentrations. The *umu*-test is based on the ability of genotoxins to induce expression of the *umuC* gene, one of the SOS genes responsible for error-prone repair; this gene is more involved in mutagenesis than the other known SOS genes in bacteria. The tester strain (*S. typhimurium* TA1535/pSK1002) carries a fused *umuC*–*lacZ* gene, allowing the monitoring of *umuC* expression by measuring β-galactosidase activity in a colorimetric assay. The *umu*-test can detect many types of DNA-damaging agents and can be used for the screening of amino acid- and nutrient-containing samples such as urine, serum, and food compounds [57].

### RNA preparation and quantitative real-time PCR of colonic tissues

Frozen middle colonic tissues were processed for total RNA preparation with TaKaRa FastPure RNA Kits (Takara) with on-column DNase treatment following the manufacturer's protocol. Quantity, purity, and integrity were confirmed initially by spectrophotometry and electrophoresis using an Agilent Bioanalyzer 2100 (Agilent Technologies, Palo Alto, CA, USA).

### Quantitative real-time PCR of colonic gene expression

cDNA for each sample was synthesized using 200 ng total RNA and PrimeScript RT reagent Kits, according to the manufacturer's instructions (Takara). Real-time PCR was performed with a StepOne Real-Time PCR System (Applied Biosystems) with TaqMan Fast Universal PCR Master Mix (Applied Biosystems) using TaqMan probes (Tnf: Mm99999068_m1, Traf6: Mm00493836_m1, Muc2: Mm00458299_m1, and β-actin: Mm02619580_g1) purchased as TaqMan Gene Expression Assays (Applied Biosystems). For occludin and ZO-1, real-time PCR was performed with a StepOne Real-Time PCR System with SYBR Green PCR Master Mix (Applied Biosystems). The sense and antisense primers for occludin were 5′-ATCCTGGGCATCATGGTGTTT-3′ and 5′-GGGCCGTCGGGTTCACT-3′, and those for ZO-1 were 5′-CCAGGCATCATCCCAAATAAGAA-3′ and 5′-CCACCCGCTGTCTTTGGA-3′. Amplifications were performed using the following temperature profiles: 1 cycle at 95°C for 30 s, 40 cycles of denaturation at 95°C for 15 s, annealing at the optimal temperature (occludin, 65°C; ZO-1, 64°C) for 30 s, and elongation at 72°C for 30 s. Melting curves were obtained by heating samples from optimal temperature to 95°C in increments of 0.1°C per s, with continuous fluorescence collection. The comparative delta Ct method was used for normalizations with the housekeeping gene β-actin.

### Microarray analysis of gene expression

RNA was extracted from the middle colon of 3 mice per group. DNA microarray was performed by using the hybridized RNA from each group. Changes in gene expression levels were evaluated using the NimbleGen mouse DNA microarray (25,631 different mouse genes including 3 probes consisting of 60-mer synthetic oligonucleotides for each gene). All hybridizations, staining, and processing were performed by personnel at Roche NimbleGen, Inc. (Madison, WI, USA).

Expression data were normalized using GeneSpring version GX (Agilent Technologies, Inc., Santa Clara, CA, USA). Gene annotation information was obtained from NCBI (http://www.ncbi.nlm.nih.gov/) and Mouse Genome Informatics (MGI) (http://www.informatics.jax.org/). To identify what biological activities were modulated by the LKM512 treatment, we performed gene clustering using the L2L Microarray Analysis Tool (http://depts.washington.edu/l2l/about.html). Gene pathway information was obtained from GenMAPP (http://www.genmapp.org/). The pathway set was tested for Gene set enrichment using Parametric analysis of Gene set enrichment (PAGE) [58]. The raw data has been deposited in a MIAME compliant database, CIBEX (Center for Information Biology Gene Expression database: http://cibex.nig.ac.jp/index.jsp) (accession number: CBX171).

### Histological analysis

Proximal colon samples used for histological analysis were fixed with 15% neutral formalin, embedded in paraffin, sectioned at 3 µm, and stained with hematoxylin 3G and eosin (Sakura Finetek Japan, Tokyo, Japan) for HE staining and with Schiff's Reagent (Sigma-Aldrich, Inc., St. Louis, MO, USA) for periodic acid-Schiff (PAS) staining.

### Statistical analysis

Survival curves were drawn using the Kaplan–Meier method and the survival rate was compared with the Log-rank test. The markers for each experimental mouse group were compared using Mann-Whitney U tests. The incidence of skin ulcers and visible tumors was assayed by Fisher's exact tests. StatMate IV (ATMS Co. Ltd., Tokyo, Japan) was used to conduct all statistical analyses.

## Supporting Information

Figure S1 **Correlation between fecal spermine concentration and 16S rRNA gene expression for the predominant intestinal bacterial group.**
(PPT)

Figure S2 **Comparison of pathways up- (red) and downregulated (blue) by LKM512 administration and ageing (All pathways).** |Z-score| more than 1.98 was considered significant.
(PPT)

Figure S3 **Microarray data scatter plots of the genes involved in the inflammatory cytokines pathway.** All genes (n = 25,631) are displayed on scatter plots. The values for all the genes of the IL-1 (upper), IL-2 (middle), and IL-6 pathways (bottom) represented on the array are highlighted in black. Younger (pretreatment) vs. control (left), LKM512 vs. control (middle), and LKM512 vs. younger mice (right).
(PPT)

Figure S4 **Effects of LKM512 on oxidative stress.** (*A*) Urinary 8-OHdG concentrations in the 3 treatment groups. (*B*) Microarray data scatter plots of genes involved in the oxidative stress pathway. All genes (n = 25,631) are represented on scatter plots. The values for all the oxidative stress pathway genes represented on the array are highlighted in black. Younger vs. control (left), LKM512 vs. control (middle), and LKM512 vs. younger mice (right).
(PPT)

Figure S5 **Effects of oral SPM administration on mice.** (*A*) Kaplan–Meier survival curves for mice in the SPM groups. Mice treated with SPM also tended to live longer than controls (*P* = 0.096), but this difference was not significant. Additionally, LKM512-treated mice tended to live longer than SPM-treated mice (*P* = 0.121). (*B*) Fecal SPM concentrations in SPM-treated mice compared to those in other groups. Fecal SPM concentrations in SPM mice were lower than those in control mice, supporting previous observations that exogenous PAs derived from meals are absorbed before reaching the lower parts of the intestine. (*C*) Hierarchical clustering showing the relationship between the patterns of expression among SPM-treated and other mice. Red and green indicate up- and downregulation of gene expression, respectively. Expression patterns in LKM512 mice were similar to those in younger mice, and expression patterns of SPM-treated mice were similar to those in control mice; however, the patterns of these 2 pairs of groups contrasted with each other. (*D*) Incidence of skin ulcers and visible tumors in the SPM-treated mice and other groups. Among mice in the SPM-treated group, the incidence of skin ulcers and visible tumors was lower than that in the control group (*P*<0.05).
(PPT)

Figure S6 **Colonic autophagy was noted to be induced by LKM512 treatment.** Lysates of colonic tissue derived from 21-month-old LKM512 mice and control mice were subjected to immunoblot analysis with an anti-LC3 antibody. The positions of β-actin as a positive control, LC3-I, and LC3-II are indicated here. The LC3-I/LC3-II ratio in the LKM512-treated mice was lower than that in control mice.
(PPT)

Table S1 **Changes in colonic gene expression in the ageing of rats, ageing of mice, and mice treated with LKM512.**
(DOC)

Table S2 **Comparison of the genes downstream of the mTOR pathway and IGF-1, AMPK, and PGC-1α gene expression in the colon among LKM512, control, and younger mice.**
(DOC)

Table S3 **Primer sets used for real-time PCR.**
(DOC)
