# Supplementary material for: Longevity in Mice Is Promoted by Probiotic-Induced Suppression of Colonic Senescence Dependent on Upregulation of Gut Bacterial Polyamine Production
Source: PLoS One. 2011 Aug 16;6(8):e23652. doi: 10.1371/journal.pone.0023652 (PMC3156754; doi:10.1371/journal.pone.0023652)
Supplement: Figure S1 — Correlation between fecal spermine concentration and 16S rRNA gene expression for the predominant intestinal bacterial group. (PPT) [file pone.0023652.s001.ppt]

## Slide 1
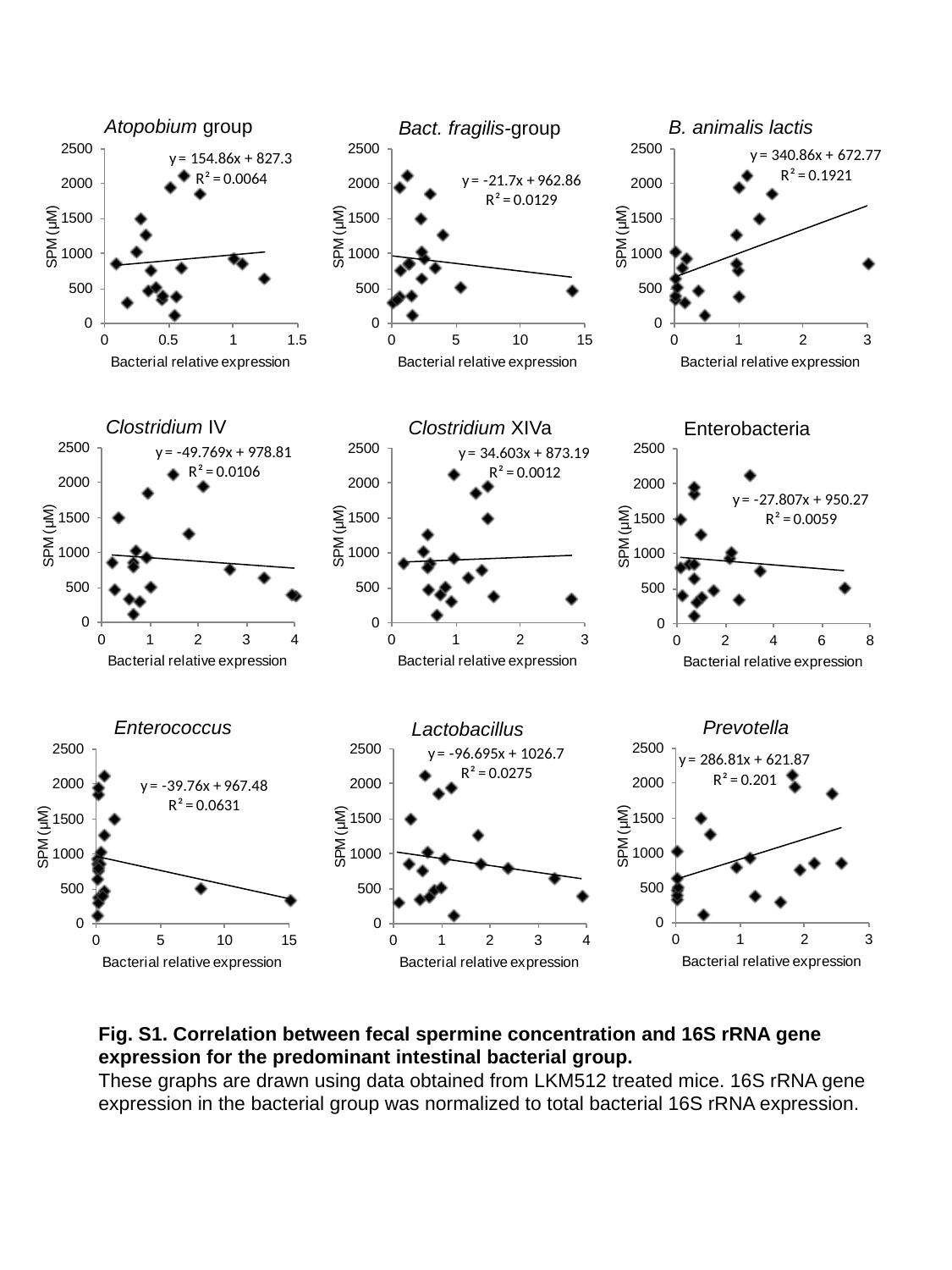

Atopobium group
B. animalis lactis
Bact. fragilis-group
Clostridium IV
Clostridium XIVa
Enterobacteria
Enterococcus
Prevotella
Lactobacillus
Fig. S1. Correlation between fecal spermine concentration and 16S rRNA gene expression for the predominant intestinal bacterial group.
These graphs are drawn using data obtained from LKM512 treated mice. 16S rRNA gene expression in the bacterial group was normalized to total bacterial 16S rRNA expression.
