# Supplementary material for: Longevity in Mice Is Promoted by Probiotic-Induced Suppression of Colonic Senescence Dependent on Upregulation of Gut Bacterial Polyamine Production
Source: PLoS One. 2011 Aug 16;6(8):e23652. doi: 10.1371/journal.pone.0023652 (PMC3156754; doi:10.1371/journal.pone.0023652)
Supplement: Figure S2 — Comparison of pathways up- (red) and downregulated (blue) by LKM512 administration and ageing (All pathways). |Z-score| more than 1.98 was considered significant. (PPT) [file pone.0023652.s002.ppt]

## Slide 1
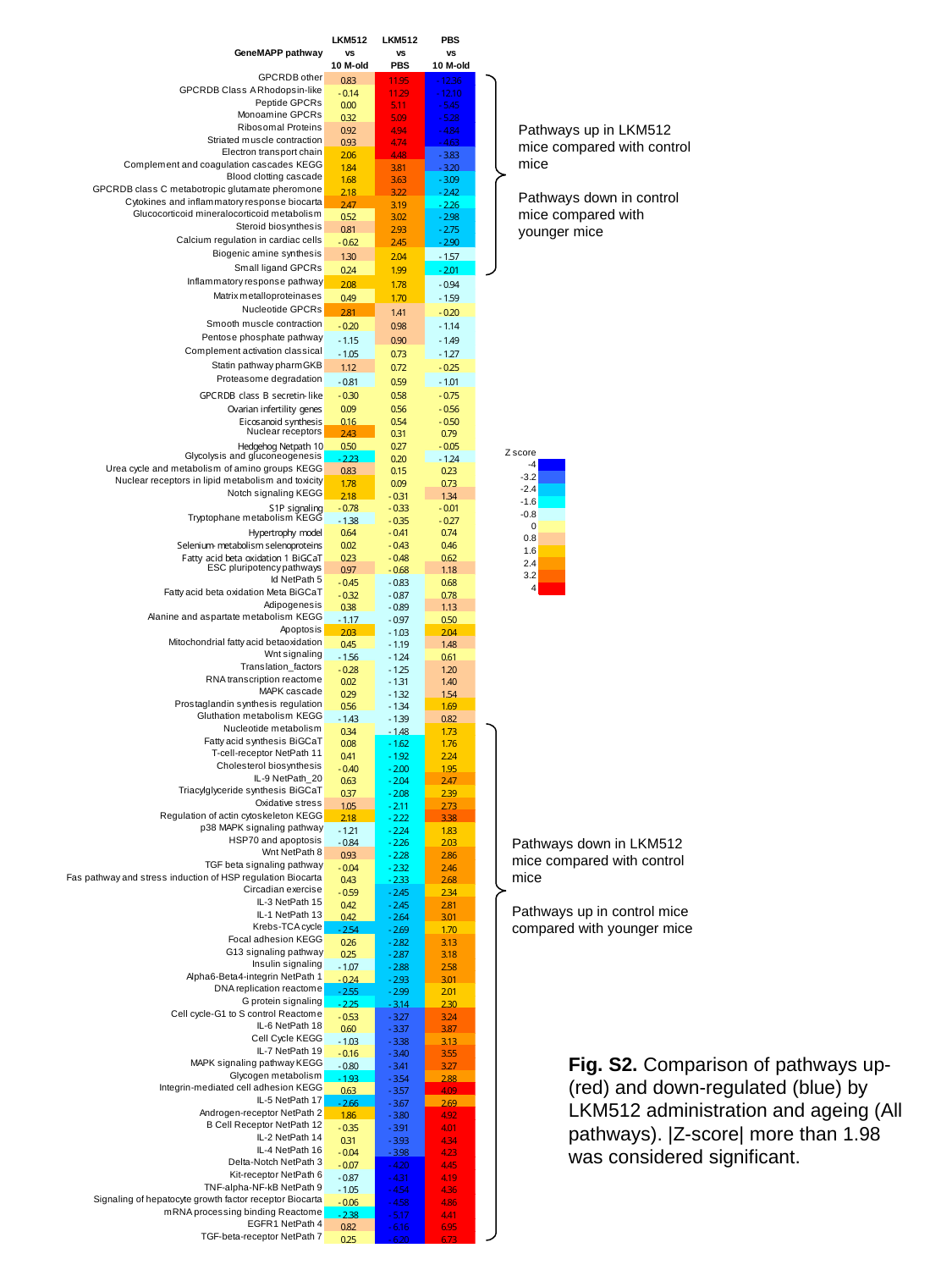

Pathways up in LKM512 mice compared with control mice
Pathways down in control mice compared with younger mice
Pathways down in LKM512 mice compared with control mice
Pathways up in control mice compared with younger mice
Fig. S2. Comparison of pathways up- (red) and down-regulated (blue) by LKM512 administration and ageing (All pathways). |Z-score| more than 1.98 was considered significant.
