# Supplementary material for: Longevity in Mice Is Promoted by Probiotic-Induced Suppression of Colonic Senescence Dependent on Upregulation of Gut Bacterial Polyamine Production
Source: PLoS One. 2011 Aug 16;6(8):e23652. doi: 10.1371/journal.pone.0023652 (PMC3156754; doi:10.1371/journal.pone.0023652)
Supplement: Figure S3 — Microarray data scatter plots of the genes involved in the inflammatory cytokines pathway. All genes (n = 25,631) are displayed on scatter plots. The values for all the genes of the IL-1 (upper), IL-2 (middle), and IL-6 pathways (bottom) represented on the array are highlighted in black. Younger (pretreatment) vs. control (left), LKM512 vs. control (middle), and LKM512 vs. younger mice (right). (PPT) [file pone.0023652.s003.ppt]

## Slide 1
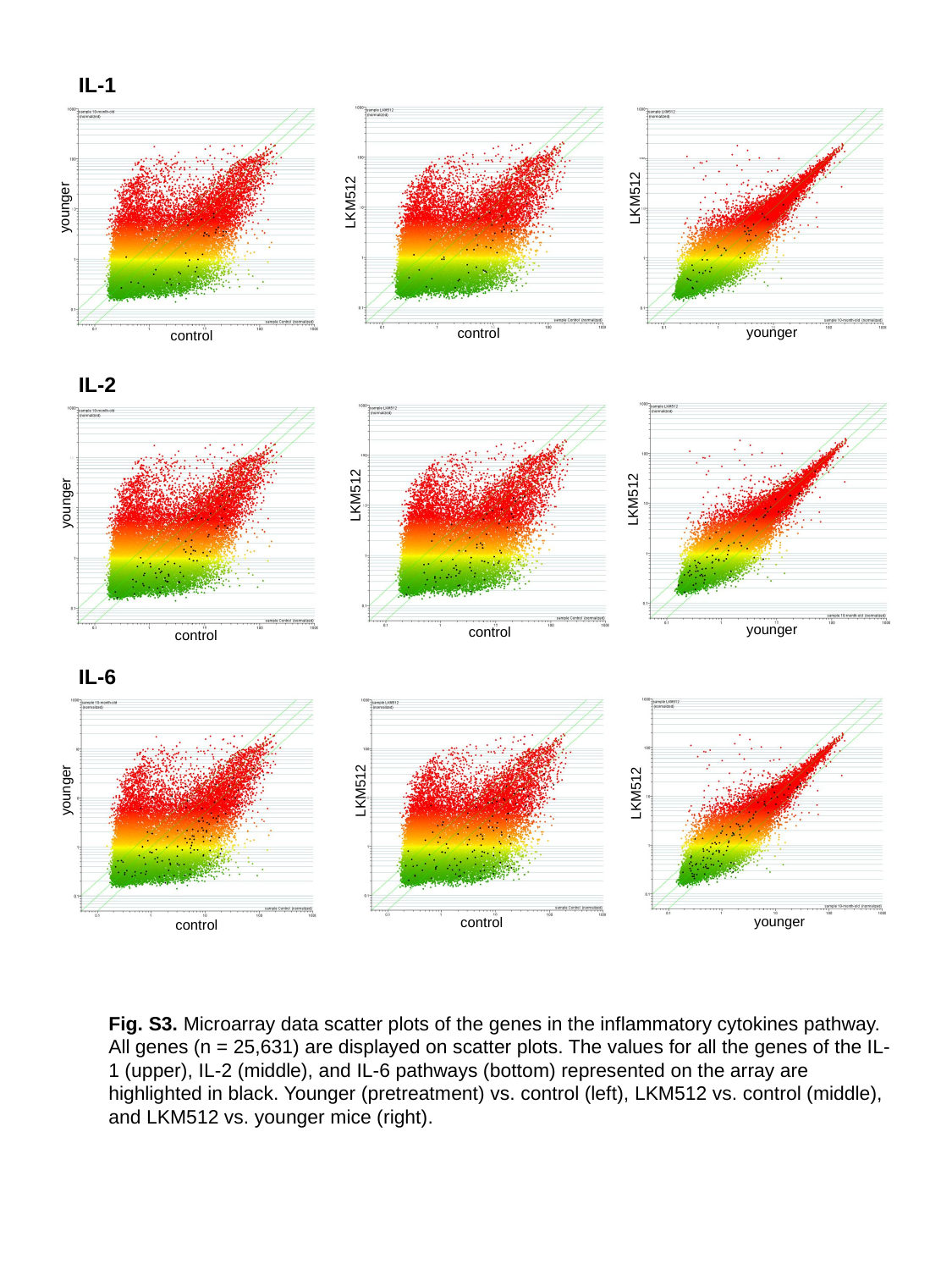

IL-1
LKM512
LKM512
younger
younger
control
control
IL-2
LKM512
younger
LKM512
younger
control
control
IL-6
younger
LKM512
LKM512
younger
control
control
Fig. S3. Microarray data scatter plots of the genes in the inflammatory cytokines pathway. All genes (n = 25,631) are displayed on scatter plots. The values for all the genes of the IL-1 (upper), IL-2 (middle), and IL-6 pathways (bottom) represented on the array are highlighted in black. Younger (pretreatment) vs. control (left), LKM512 vs. control (middle), and LKM512 vs. younger mice (right).
