# Supplementary material for: Longevity in Mice Is Promoted by Probiotic-Induced Suppression of Colonic Senescence Dependent on Upregulation of Gut Bacterial Polyamine Production
Source: PLoS One. 2011 Aug 16;6(8):e23652. doi: 10.1371/journal.pone.0023652 (PMC3156754; doi:10.1371/journal.pone.0023652)
Supplement: Figure S4 — Effects of LKM512 on oxidative stress. (A) Urinary 8-OHdG concentrations in the 3 treatment groups. (B) Microarray data scatter plots of genes involved in the oxidative stress pathway. All genes (n = 25,631) are represented on scatter plots. The values for all the oxidative stress pathway genes represented on the array are highlighted in black. Younger vs. control (left), LKM512 vs. control (middle), and LKM512 vs. younger mice (right). (PPT) [file pone.0023652.s004.ppt]

## Slide 1
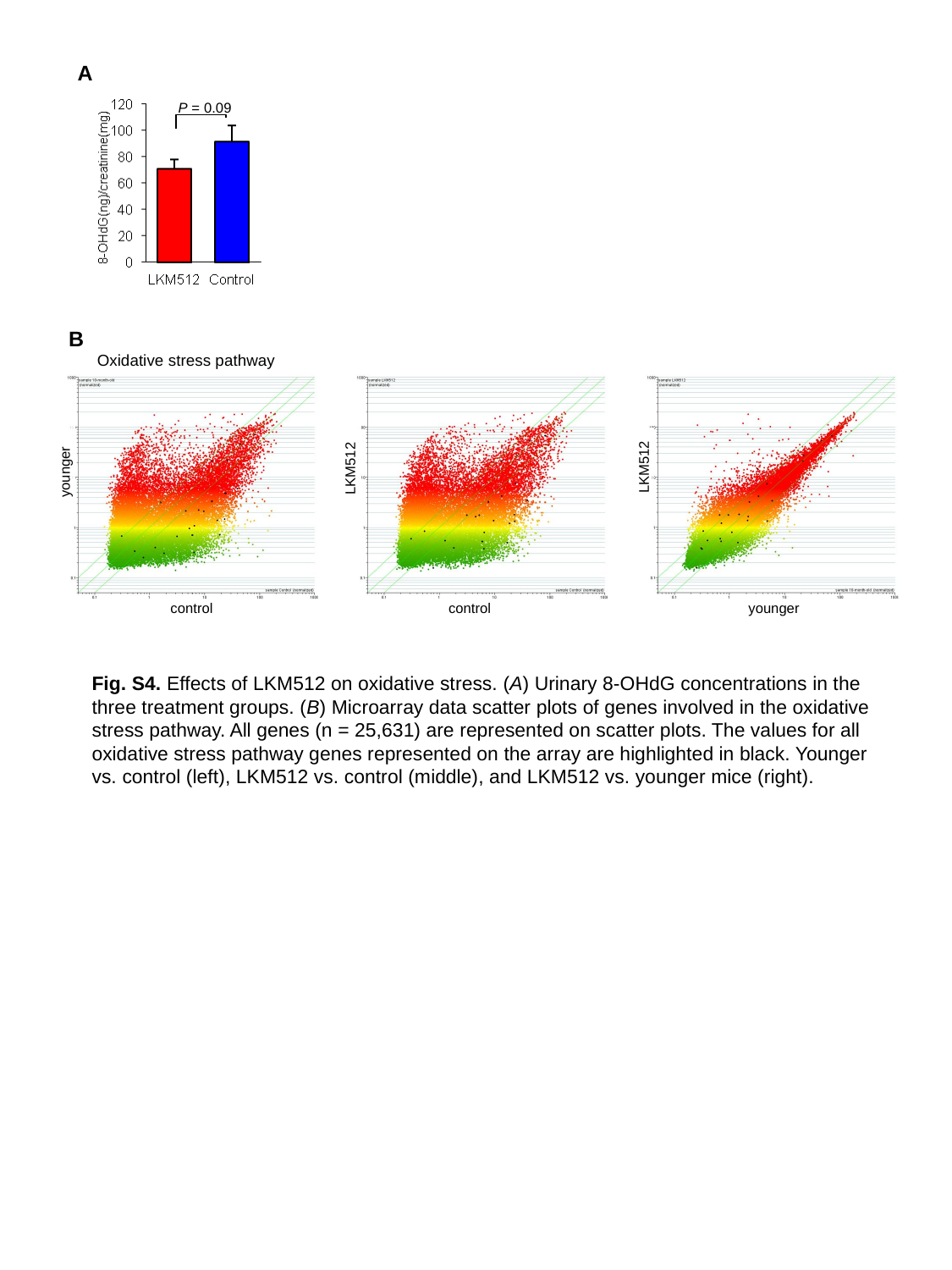

A
P = 0.09
B
 Oxidative stress pathway
younger
LKM512
LKM512
control
control
younger
Fig. S4. Effects of LKM512 on oxidative stress. (A) Urinary 8-OHdG concentrations in the three treatment groups. (B) Microarray data scatter plots of genes involved in the oxidative stress pathway. All genes (n = 25,631) are represented on scatter plots. The values for all oxidative stress pathway genes represented on the array are highlighted in black. Younger vs. control (left), LKM512 vs. control (middle), and LKM512 vs. younger mice (right).
