# Supplementary material for: Longevity in Mice Is Promoted by Probiotic-Induced Suppression of Colonic Senescence Dependent on Upregulation of Gut Bacterial Polyamine Production
Source: PLoS One. 2011 Aug 16;6(8):e23652. doi: 10.1371/journal.pone.0023652 (PMC3156754; doi:10.1371/journal.pone.0023652)
Supplement: Figure S5 — Effects of oral SPM administration on mice. (A) Kaplan–Meier survival curves for mice in the SPM groups. Mice treated with SPM also tended to live longer than controls (P = 0.096), but this difference was not significant. Additionally, LKM512-treated mice tended to live longer than SPM-treated mice (P = 0.121). (B) Fecal SPM concentrations in SPM-treated mice compared to those in other groups. Fecal SPM concentrations in SPM mice were lower than those in control mice, supporting previous observations that exogenous PAs derived from meals are absorbed before reaching the lower parts of the intestine. (C) Hierarchical clustering showing the relationship between the patterns of expression among SPM-treated and other mice. Red and green indicate up- and downregulation of gene expression, respectively. Expression patterns in LKM512 mice were similar to those in younger mice, and expression patterns of SPM-treated mice were similar to those in control mice; however, the patterns of these 2 pairs of groups contrasted with each other. (D) Incidence of skin ulcers and visible tumors in the SPM-treated mice and other groups. Among mice in the SPM-treated group, the incidence of skin ulcers and visible tumors was lower than that in the control group (P<0.05). (PPT) [file pone.0023652.s005.ppt]

## Slide 1
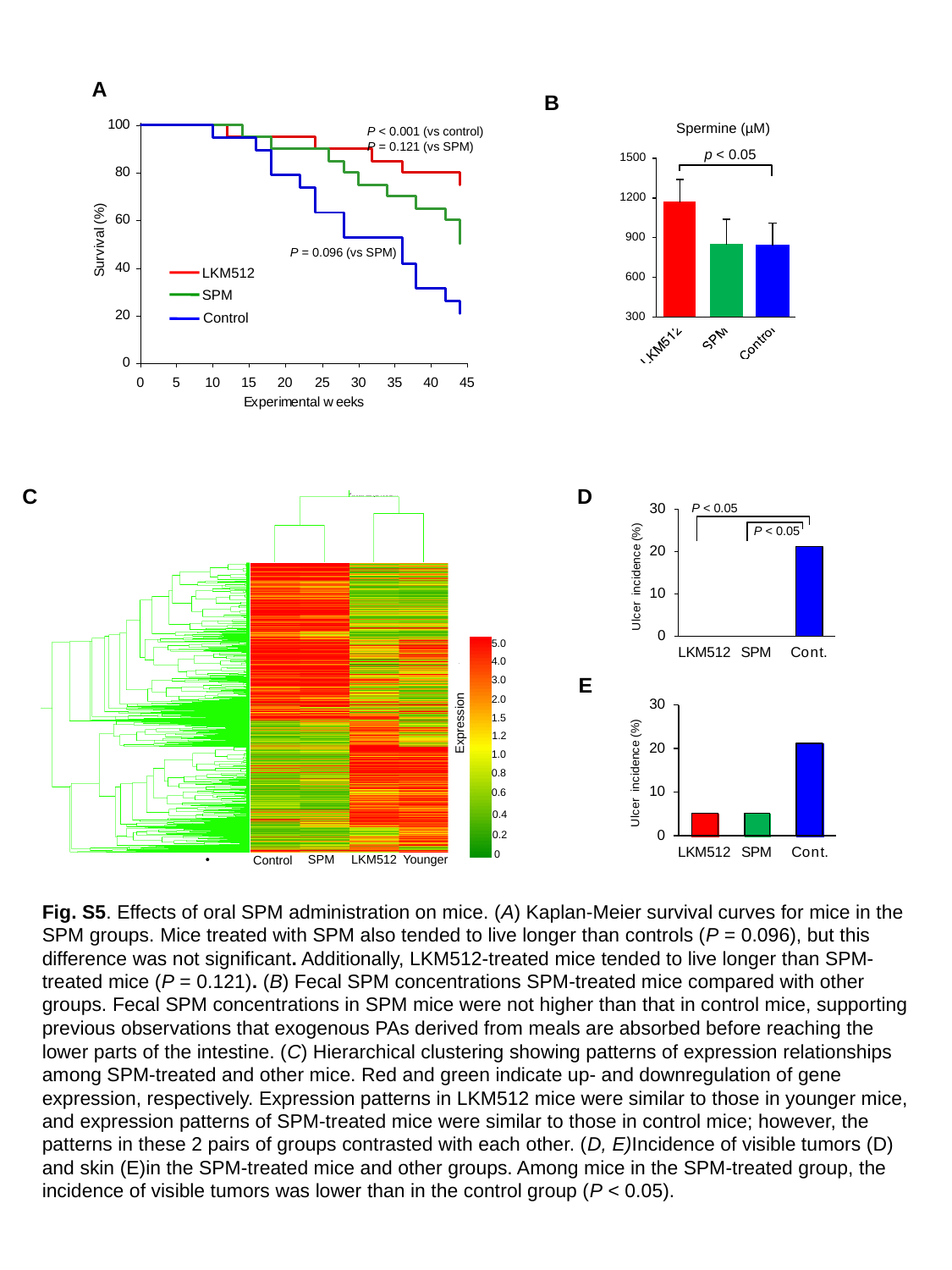

A
B
Spermine (µM)
P < 0.001 (vs control)
P = 0.121 (vs SPM)
p < 0.05
P = 0.096 (vs SPM)
LKM512
SPM
Control
C
D
P < 0.05
P < 0.05
5.0
4.0
E
3.0
Expression
2.0
1.5
1.2
1.0
0.8
0.6
0.4
0.2
0
SPM
LKM512
Younger
Control
Fig. S5. Effects of oral SPM administration on mice. (A) Kaplan-Meier survival curves for mice in the SPM groups. Mice treated with SPM also tended to live longer than controls (P = 0.096), but this difference was not significant. Additionally, LKM512-treated mice tended to live longer than SPM-treated mice (P = 0.121). (B) Fecal SPM concentrations SPM-treated mice compared with other groups. Fecal SPM concentrations in SPM mice were not higher than that in control mice, supporting previous observations that exogenous PAs derived from meals are absorbed before reaching the lower parts of the intestine. (C) Hierarchical clustering showing patterns of expression relationships among SPM-treated and other mice. Red and green indicate up- and downregulation of gene expression, respectively. Expression patterns in LKM512 mice were similar to those in younger mice, and expression patterns of SPM-treated mice were similar to those in control mice; however, the patterns in these 2 pairs of groups contrasted with each other. (D, E)Incidence of visible tumors (D) and skin (E)in the SPM-treated mice and other groups. Among mice in the SPM-treated group, the incidence of visible tumors was lower than in the control group (P < 0.05).
