# Supplementary material for: Longevity in Mice Is Promoted by Probiotic-Induced Suppression of Colonic Senescence Dependent on Upregulation of Gut Bacterial Polyamine Production
Source: PLoS One. 2011 Aug 16;6(8):e23652. doi: 10.1371/journal.pone.0023652 (PMC3156754; doi:10.1371/journal.pone.0023652)
Supplement: Figure S6 — Colonic autophagy was noted to be induced by LKM512 treatment. Lysates of colonic tissue derived from 21-month-old LKM512 mice and control mice were subjected to immunoblot analysis with an anti-LC3 antibody. The positions of β-actin as a positive control, LC3-I, and LC3-II are indicated here. The LC3-I/LC3-II ratio in the LKM512-treated mice was lower than that in control mice. (PPT) [file pone.0023652.s006.ppt]

## Slide 1
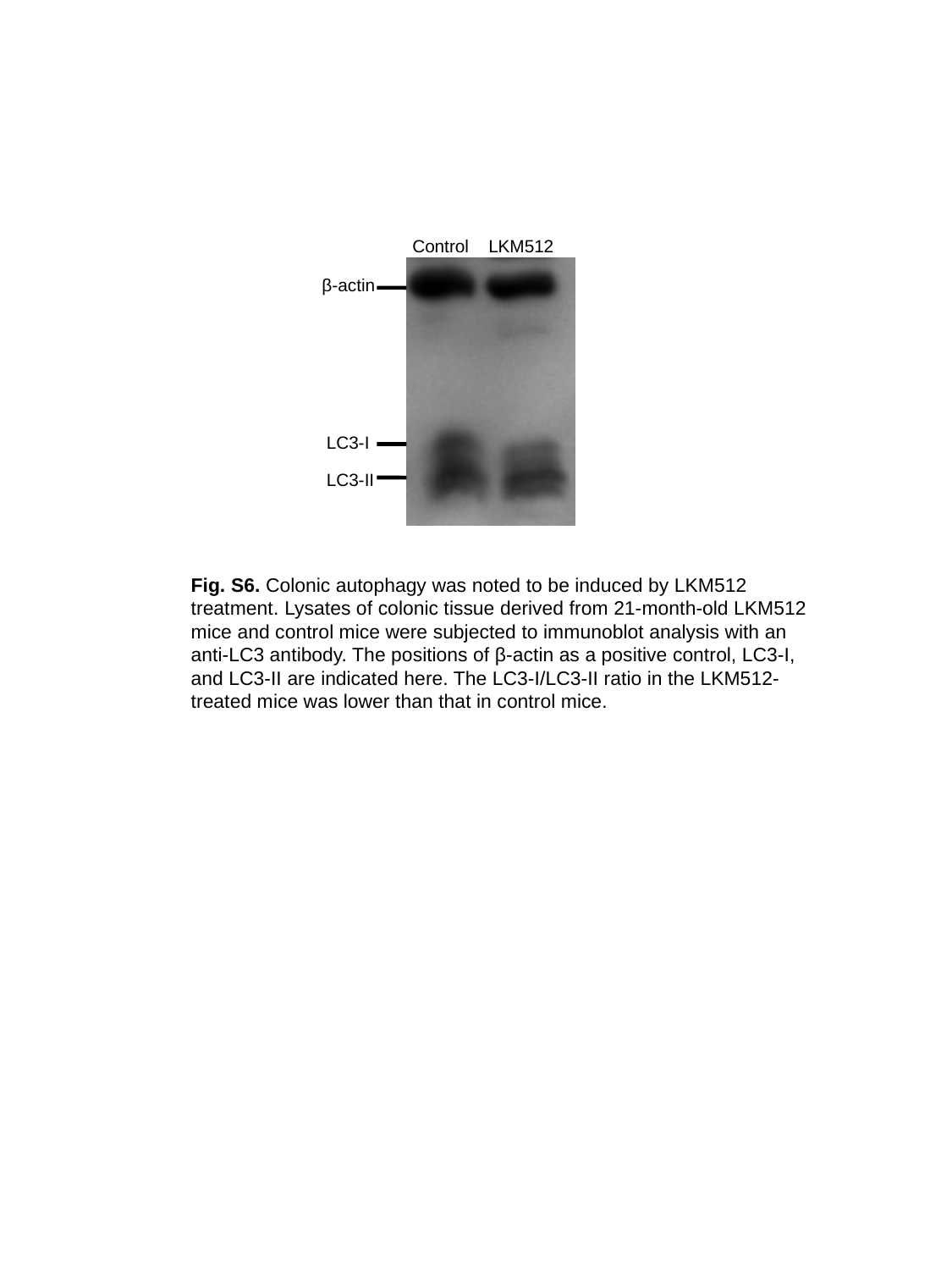

Control
LKM512
β-actin
LC3-I
LC3-II
Fig. S6. Colonic autophagy was noted to be induced by LKM512 treatment. Lysates of colonic tissue derived from 21-month-old LKM512 mice and control mice were subjected to immunoblot analysis with an anti-LC3 antibody. The positions of β-actin as a positive control, LC3-I, and LC3-II are indicated here. The LC3-I/LC3-II ratio in the LKM512-treated mice was lower than that in control mice.
