# Supplementary material for: Longevity in Mice Is Promoted by Probiotic-Induced Suppression of Colonic Senescence Dependent on Upregulation of Gut Bacterial Polyamine Production
Source: PLoS One. 2011 Aug 16;6(8):e23652. doi: 10.1371/journal.pone.0023652 (PMC3156754; doi:10.1371/journal.pone.0023652)
Supplement: Table S3 — Primer sets used for real-time PCR. (DOC) [file pone.0023652.s009.doc]

| **Table S3.** Primer sets used for real-time PCR | |  |
| --- | --- | --- |
| Target | Sequence(5' to 3') | Annealing  temp (ºC) |
| *B. animalis* subsp. *lactis* | F: CCCTTTCCACGGGTCCC | 65 |
|  | R: AAGGGAAACCGTGTCTCCAC |  |
| *Clostridium* subcluster XVIa | F: AAATGACGGTACCTGACTAA | 50 |
|  | R: CTTTGAGTTTCATTCTTGCGAA |  |
| *Clostridium* cluster IV | F: GCACAAGCAGTGGAGT | 50 |
|  | R: AGTSCTCTTGCGTAG |  |
| *Bacteroides fragilis* group | F: ATAGCCTTTCGAAAGRAAGAT | 50 |
|  | R: CCAGTATCAACTGCAATTTTA |  |
| *Prevotella* spp. | F: CACRGTAAACGATGGATGCC | 55 |
|  | R: GGTCGGGTTGCAGACC |  |
| *Atopobium* group | F: ACCGCTTTCAGCAGGGA | 60 |
|  | R: ACGCCCAATGAATCCGGAT |  |
| *Lactobacillus* group | F: AGCAGTAGGGAATCTTCCA | 58 |
|  | R: CACCGCTACACATGGAG |  |
| Enterobacteriaceae | F: TGCCGTAACTTCGGGAGAAGGCA | 60 |
|  | R: TCAAGGCTCAATGTTCAGTGTC |  |
| *Enterococcus* spp. | F: CCCTTATTGTTAGTTGCCATCATT | 61 |
|  | R: ACTCGTTGTACTTCCCATTGT |  |
| Total bacteria | F: TCCTACGGGAGGCAGCAGT | 60 |
|  | R: GGACTACCAGGGTATCTAATCCTGTT |  |
