# Supplementary material for: Longevity in Mice Is Promoted by Probiotic-Induced Suppression of Colonic Senescence Dependent on Upregulation of Gut Bacterial Polyamine Production
Source: PLoS One. 2011 Aug 16;6(8):e23652. doi: 10.1371/journal.pone.0023652 (PMC3156754; doi:10.1371/journal.pone.0023652)
Supplement: Supplementary file 8 [file pone.0023652.s007.doc]

Table S1. Changes in colonic gene expression in the ageing of rats, ageing of mice, and mice treated with LKM512

Systematic Name	Fold change: Rat Colon[1] Ageing vs Young	Fold change: This study Control vs Younger	This study LKM512 vs Control	This study LKM512 vs Younger	GenBank[2]
Cell surface antigens					
annexin A5	2.1	1.726	0.467	0.805	AK153476
CD44 antigen	3.0	4.928	0.111	0.546	X66081
cadherin 17	2.2	4.522	0.139	0.628	BC042891
CD24a antigen	2.5	1.871	0.528	0.987	BC026702
peripheral myelin protein	2.8	2.543	0.322	0.819	AK171745
serpin B5	2.3	6.158	0.115	0.709	AK136532
Cell cycle and nuclear proteins					
proliferating cell nuclear antigen	3.0	1.513	0.44	0.666	AK168233
cyclin D1	2.3	0.854	1.045	0.893	BC044841
high mobility group box 2	13.0	1.797	0.66	1.187	AK151164
high mobility group box 1	4.0	1.622	0.569	0.923	Z11997
heterogeneous nuclear ribonucleoprotein K	3.5	2.583	0.227	0.585	BC006694
Nutrient digestion and absorption					
solute carrier family 13 member 3	2.0	2.252	0.331	0.745	AF201903
ATPase, Na+/K+ transporting, beta 1 polypeptide	3.2	3.516	0.127	0.446	X61433
fatty acid binding protein 2, intestinal	3.3	1.427	0.873	1.245	AY523819
valosin containing protein	2.5	1.093	0.618	0.676	AK207315
colipase, pancreatic	0.16	7.099	1.121	7.961	AK008874
Signal transduction					
RAB1, member RAS oncogene family	2.9	2.54	0.355	0.902	AK167150
guanosine diphosphate (GDP) dissociation inhibitor 2	6.1	3.64	0.117	0.428	U07951
GTPase KRas	3.0	10.83	0.0529	0.573	BC119495
mitogen-activated protein kinase (MAPK)1	2.4	6.21	0.118	0.735	D10939
endothelial differentiation, sphingolipid G-protein-coupled receptor, 5	3.1	0.703	1.058	0.744	AK196049
PDGFA associated protein 1	0.14	0.667	0.82	0.547	BC055927
calmodulin 3	3.0	1.65	0.656	1.083	AK153179
Intercellular signaling					
sulfotransferase family 1B, member 1	4.6	11.14	0.0793	0.884	NM_019878
annexin A2	2.1	2.215	0.296	0.655	AK165888
cholinergic receptor, nicotinic, gamma polypeptide	0.42	0.256	1.045	0.267	NM_009604
5 hydroxytryptamine (serotonin) receptor 4	0.45	4.14	0.295	1.221	BC153066
insulin-like growth factor binding protein 3	0.50	2.668	1.191	3.179	AK077477
solute carrier family 6 (neurotransmitter transporter, serotonin), member 4	5.2	0.931	0.989	0.92	BC108978
vesicle-associated membrane protein 2	2.1	3.779	0.286	1.079	AK090178
caspase 1	3.2	7.059	0.144	1.016	L03799
Energy matabolism					
ADP/ATP translocase 2	2.0	1.673	0.528	0.884	BC004570
aldolase 1, A isoform	2.4	1.457	0.619	0.902	EF662059
acyl-Coenzyme A dehydrogenase, medium chain	41.0	6.081	0.128	0.778	U07159
ATP synthase, H+ transporting, mitochondrial F0 complex, subunit d	3.9	0.656	1.788	1.173	BC081431
ATP synthase, H+ transporting mitochondrial F1 complex, beta subunit	2.6	2.18	0.478	1.043	AK198338
adenylate kinase 3	2.2	22.88	0.0607	1.388	BC024871
ATP synthase, H+ transporting, mitochondrial F0 complex, subunit c (subunit 9),	2.3	0.543	1.023	0.555	AK008191
Metabolic enzyme					
alcohol dehydrogenase 1 (class I)	6.1	1.246	0.72	0.897	AK146094
hydroxy-delta-5-steroid dehydrogenase, 3 beta- and steroid delta-isomerase 6	4.6	0.648	1.408	0.912	BC053501
cytochrome P450, family 4, subfamily f, polypeptide 14	2.3	1.586	1.817	2.882	AK018676
glutathione synthetase	6.9	3.822	0.236	0.903	AK159630
acyl-Coenzyme A dehydrogenase, long-chain	2.4	3.075	0.21	0.645	AK184517
aldo-keto reductase family 1, member A4 (aldehyde reductase)	2.4	1.274	0.574	0.731	BC039926
phosphatidic acid phosphatase 2a	62.0	2.561	0.489	1.252	D84376
acyl-CoA thioesterase 7	8.2	2.559	0.521	1.333	AK198843
Protein metabolism					
ubiquitin-conjugating enzyme E2B, RAD6 homology (S. cerevisiae)	19.0	3.466	0.0474	0.164	AK153174
ribosomal protein S4, X-linked	52.0	1.028	0.951	0.977	AK153211
ribosomal protein L11	2.5	0.782	1.247	0.975	AK014593
ribosomal protein L13	2.0	0.673	1.506	1.013	AK202315
ribosomal protein L21	2.1	1.853	0.649	1.202	BC096572
ribosomal protein S11	3.2	1.113	0.879	0.978	AK150738
ribosomal protein S17	6.0	0.798	1.14	0.909	D25213
eukaryotic translation initiation factor 2, subunit 1 alpha	2.4	4.633	0.205	0.948	AK049618
Cytoskeletal proteins					
cofilin 1, non-muscle	2.0	2.131	1.393	2.967	BC058726
calponin 1	3.2	0.303	1.355	0.411	AK035548

Numbers show fold increase.
Genes altered by ageing in the rats are listed.
[1] This data is cited from the article by Lee et al. (24).
[2] Gene bank numbers are for mice gene.
