# Supplementary material for: Longevity in Mice Is Promoted by Probiotic-Induced Suppression of Colonic Senescence Dependent on Upregulation of Gut Bacterial Polyamine Production
Source: PLoS One. 2011 Aug 16;6(8):e23652. doi: 10.1371/journal.pone.0023652 (PMC3156754; doi:10.1371/journal.pone.0023652)
Supplement: Supplementary file 9 [file pone.0023652.s008.doc]

Table S2. Comparison of the genes downstream of the mTOR pathway and IGF-1, AMPK, and PGC-1α gene expression in the colon among LKM512, control, and younger mice

	Fold change			
	LKM512 vs. Younger	Control vs. Younger	LKM512 vs. Control	GenBank
Genes located downstream of mTORC				
FK506 binding protein 12-rapamycin associated protein 1	0.901	1.03	0.874	BC021454
G protein beta subunit-like	4.093	2.382	1.718	AK077680
regulatory associated protein of MTOR, complex 1	0.909	9.343	0.0973	AK082586
hypoxia inducible factor 1, alpha subunit	0.622	10.49	0.0592	BC026139
ribosomal protein S6 kinase, polypeptide 1	0.913	1.241	0.736	AK053930
ribosomal protein S6 kinase, polypeptide 2	1.206	0.856	1.41	BC066842
eukaryotic translation initiation factor 4E binding protein 1	1.315	1.764	0.745	BE651550
Unc-51 like kinase 1 (C. elegans)	2.566	8.595	0.299	BC023281
Unc-51 like kinase 2 (C. elegans)	1.331	2.357	0.565	AK146620
unc-51-like kinase 3 (C. elegans)	1.334	0.344	3.877	BC037093
insulin-like growth factor 1	3.362	4.181	0.804	AY878192
protein kinase, AMP-activated, alpha 1 catalytic subunit	0.346	6.057	0.0571	CJ084953
protein kinase, AMP-activated, gamma 1 non-catalytic subunit	0.861	2.742	0.314	AK159583
protein kinase, AMP-activated, beta 1 non-catalytic subunit	0.672	8.726	0.077	AK088250
protein kinase, AMP-activated, gamma 2 non-catalytic subunit	0.758	8.761	0.0865	AK165834
protein kinase, AMP-activated, gamma 3 non-catatlytic subunit	0.405	0.355	1.141	AF525501
protein kinase, AMP-activated, alpha 2 catalytic subunit	0.552	17.83	0.031	BY065557
protein kinase, AMP-activated, beta 2 non-catalytic subunit	1.203	4.305	0.28	AK037124
peroxisome proliferative activated receptor, gamma, coactivator 1 alpha	0.751	7.452	0.101	AK138668

Numbers show fold increase or decrease.
